# Hidden low wellbeing among high-achieving students: model-based support-cost patterns revealed by explainable machine learning

**DOI:** 10.3389/fpsyg.2026.1891921

**Published:** 2026-08-25

**Authors:** Xinghong Hu, Renzhi Lin, Shaodong Tang

**Affiliations:** 1 Chongqing Vocational Institute of Engineering, Chongqing, China; 2 College of Education, Hunan University of Science and Technology, Xiangtan, Hunan, China

**Keywords:** explainable machine learning, life satisfaction, relatively high-achieving students, school belonging, support-cost predictive patterns

## Abstract

**Introduction:**

High achievement is often treated as evidence of positive adjustment, yet some relatively high-achieving students continue to perform well while reporting low subjective wellbeing. Guided by Self-Determination Theory and Expectancy-Value Theory, this study examined whether relatively high-achieving students with low and high life satisfaction could be distinguished by support-related and cost-related predictive signals.

**Methods:**

PISA 2022 data from 15 education systems were analyzed. Relatively high-achieving students were defined as those in the top 25% of mathematics performance within their education system. Life satisfaction scores of 0 to 4 indicated low life satisfaction, whereas scores of 7 to 10 indicated high life satisfaction. The final sample included 24,947 students, comprising 20,862 students with high achievement and high life satisfaction and 4,085 students with high achievement and low life satisfaction. Eleven machine-learning classifiers were compared, and the selected Gradient Boosting Machine model was interpreted using SHapley Additive exPlanations.

**Results:**

The final 12-feature model achieved a weighted validation AUC of 0.810 and an unweighted validation AUC of 0.828. School belonging was the strongest differentiating signal. Higher emotion regulation, family support, relationship quality at school, perceived safety, creative family environment, and self-directed learning efficacy generally shifted predictions toward high life satisfaction. Mathematics anxiety, bullying victimization, and self-directed learning difficulties shifted predictions toward low life satisfaction. SHAP interaction analyses indicated that low-life-satisfaction classification relied more strongly on combinations of support-related and cost-related signals centered on school belonging.

**Discussion:**

Hidden low wellbeing among relatively high-achieving students was distinguished by a life-satisfaction-based predictive profile involving relational, emotional, family, peer, and learning-experience signals. The findings may support group-level monitoring and future theory development, but they should not be interpreted as causal evidence or used as an individual diagnostic tool.

## Introduction

In educational systems where academic performance strongly shapes judgments of student success, relatively high-achieving students are often assumed to be well adjusted. Strong test scores, consistent task completion, and compliant classroom behavior may lead teachers and schools to view these students as needing less support than peers with more visible academic or behavioral difficulties (Brandmiller et al., 2020). This assumption creates an important blind spot. Some students maintain strong academic performance while experiencing low subjective wellbeing, emotional strain, or psychological discomfort. Evidence from high-pressure academic contexts suggests that strong performance does not always coincide with positive adjustment, and that some high-achieving students report anxiety, burnout, and distress despite outward success (Li and Ho, 2024; Luthar et al., 2020). Academic achievement, therefore, should not be treated as sufficient evidence of healthy adaptation.

Prior research generally reports a positive association between academic achievement and subjective wellbeing. Meta-analytic and systematic-review evidence indicates that students with higher wellbeing tend to show stronger engagement and more favorable academic outcomes, although this association is usually weak to moderate rather than strong (Amholt et al., 2020; Bücker et al., 2018). This aggregate pattern, however, does not mean that high-achieving students are a homogeneous group. Students with similar achievement levels may differ considerably in life satisfaction, school belonging, emotion regulation, perceived pressure, and exposure to adverse peer experiences. Typological and contextual studies have also shown that high achievement can coexist with psychological vulnerability, especially in competitive or high-expectation environments (Kim et al., 2015; Ng, 2020). The key issue is therefore not simply whether achievement and wellbeing are related on average, but which support-related and cost-related factors help distinguish relatively high-achieving students with high life satisfaction from those with low life satisfaction under similar performance conditions.

Existing studies have identified several correlates of student wellbeing, including school belonging, family support, peer relationships, perceived safety, anxiety, and bullying. These findings are valuable because they clarify which factors are associated with student wellbeing. They offer less insight, however, into how these factors work together to distinguish high-achieving students with low wellbeing from equally high-achieving peers with high wellbeing. Among relatively high-achieving students, low life satisfaction may not be best captured by any single predictor alone. It may instead be reflected in a predictive pattern in which lower support-related signals co-occur with higher psychological and social cost-related signals. Hidden low wellbeing can therefore be examined as a support-cost predictive pattern rather than only as a classification outcome associated with isolated risk variables.

Self-Determination Theory and Expectancy-Value Theory provide a useful basis for this interpretation. Self-Determination Theory argues that wellbeing and high-quality motivation depend on the satisfaction of autonomy, competence, and relatedness needs (Ryan and Deci, 2000, 2020). In school contexts, belonging, safety, supportive peer relationships, and family support can serve as need-supportive resources. When these resources are insufficient, students may continue to perform well while experiencing weaker relatedness, lower autonomy, or greater emotional strain (Vansteenkiste et al., 2020). Expectancy-Value Theory complements this view by emphasizing that achievement-related engagement is shaped not only by expectations and task value, but also by subjective costs such as anxiety, emotional burden, time pressure, and regulatory effort (Eccles and Wigfield, 2020; Flake et al., 2015; Muenks et al., 2023). From this perspective, some students may sustain high achievement at greater psychological cost than others. This theoretical perspective provides a basis for examining whether the coexistence of relatively high achievement and low life satisfaction is associated with lower support-related signals and higher cost-related signals.

This theoretical framing also has methodological implications. Traditional average-effect models, such as regression and structural equation modeling, are useful for estimating net associations and testing predefined pathways. The present problem, however, concerns differences within a group of students who are similarly high achieving but differ sharply in wellbeing. Such differences may involve nonlinear associations and interactions among psychological, school, family, and learning-experience indicators. Explainable machine learning is well suited to this context because it can model complex predictive patterns while preserving interpretability. In educational research, explainable artificial intelligence has increasingly been used to link predictive accuracy with interpretable evidence for decision-making and intervention planning (Khosravi et al., 2022). Recent cross-national work has also shown that machine-learning models can identify robust predictors of student wellbeing, including school belonging and parental support (King et al., 2024). Building on this literature, the present study shifts the focus from identifying general predictors of student wellbeing to examining how support-related and cost-related signals jointly distinguish hidden low life satisfaction among relatively high-achieving students.

Against this background, the present study examines hidden low wellbeing among high-achieving students through an integrated support-cost perspective. It does not approach low life satisfaction merely as a classification difference associated with isolated risk factors. Instead, it considers whether relatively high-achieving students with low life satisfaction can be distinguished by model-based combinations of lower support-related signals and higher cost-related signals. This perspective provides the basis for using explainable machine learning to identify and interpret predictive signals that differentiate relatively high-achieving students with low life satisfaction from those with high life satisfaction.

## Literature review

### The relationship between academic achievement and student wellbeing

Academic achievement is typically defined as performance in formal learning tasks and assessed using course grades, standardized test scores, credit completion, and educational progression (Scherrer et al., 2025; Vugteveen et al., 2025). In meritocracy-oriented systems, these indicators are linked to educational opportunity and social mobility, making achievement a dominant goal while diverting attention from its psychological costs (Das et al., 2022; French et al., 2024). Student wellbeing is multidimensional, encompassing physical, psychological, cognitive, and social functioning. In educational research, subjective wellbeing is often captured through affective experiences and cognitive evaluations (Pollard and Lee, 2003; Zadworna et al., 2023). Because large-scale assessments limit questionnaire length, life satisfaction is frequently used as a brief, comparable indicator of wellbeing (OECD, 2017).

Evidence links higher subjective wellbeing to better mental health, stronger engagement, and lower dropout intentions, supporting wellbeing as an educational outcome alongside achievement (Amholt et al., 2020). Meta-analyses indicate a weak-to-moderate positive association between achievement and subjective wellbeing, with an average correlation of about r = 0.16 (Bücker et al., 2018). At the same time, systematic reviews report substantial variation across studies, including null or mixed findings (Amholt et al., 2020), cautioning against assuming that higher scores reliably correspond to higher wellbeing. Longitudinal evidence suggests bidirectional relations and meaningful heterogeneity, with earlier wellbeing more consistently predicting later achievement than the reverse in some studies (Bortes et al., 2021). Typological studies also document distinct adjustment profiles in adolescence, including high achievement co-occurring with substantial distress (Kim et al., 2015).

Thus, achievement and wellbeing are positively related in aggregate, yet high achievement may accompany either high wellbeing or pronounced distress within subgroups. This motivates a profile-based predictive approach that contrasts achievement–wellbeing groups rather than relying on average associations. Here, we operationalize combinations within high achievers by comparing students with high achievement and high wellbeing to those with high achievement and low wellbeing to clarify signals differentiating a concealed risk profile.

### Motivational-theory perspective on the relationship between academic achievement and wellbeing

A single theoretical lens rarely captures the achievement–wellbeing paradox, in which students earn high grades yet feel unwell. Two influential motivation traditions, Self-Determination Theory (SDT) and Expectancy–Value Theory (EVT), approach this pattern from different angles. SDT asks whether learning environments support basic psychological needs, whereas EVT emphasizes how students weigh expected benefits against engagement costs. Together, they clarify how strong performance can coexist with compromised wellbeing.

SDT proposes that wellbeing and effective functioning are supported when autonomy, competence, and relatedness are consistently satisfied (Ryan and Deci, 2000, 2020). Contexts offering meaningful choice, appropriate challenge, clear feedback, and safe, trusting relationships are more likely to sustain intrinsic or well-internalized extrinsic motivation, enabling high achievement without sacrificing wellbeing. By contrast, settings marked by intense competition, controlling practices, and frequent social comparison may yield excellent performance while leaving students constrained, externally evaluated, and socially disconnected. Over time, these conditions can frustrate autonomy, competence, and relatedness, shift motivation toward external regulation, and heighten vulnerability to burnout, anxiety, and lower subjective wellbeing (Vansteenkiste et al., 2020). In this view, “high achievement without wellbeing” reflects how achievement is organized and pursued rather than achievement itself.

EVT explains engagement by whether effort is justified by expected returns. Participation depends on success expectations, perceived task value (e.g., importance, interest, and utility), and subjective costs (Eccles and Wigfield, 2020). EVT research emphasizes that cost, especially emotional cost and demands on time and energy, becomes salient in high-pressure contexts. When students sustain achievement through prolonged anxiety, tension, guilt, or exhaustion, motivational quality and wellbeing can erode even as performance remains high (Flake et al., 2015). From an EVT perspective, “high achievement without wellbeing” indicates that perceived benefits and meaning no longer offset cumulative learning costs.

Integrating SDT and EVT, we conceptualize the paradox as arising through two connected processes. One involves basic psychological need frustration, whereby reduced autonomy, competence, and relatedness undermine motivation and wellbeing in high-achievement environments. The other involves accumulating motivational costs, through which sustained emotional and time-related burdens contribute to emotional exhaustion and declining wellbeing. These processes likely reinforce one another: need frustration can heighten costs tied to academic pressure and social comparison, and accumulated costs can weaken felt need satisfaction. This account guides variable selection and model interpretation and motivates comparisons between students with high achievement and high wellbeing and those with high achievement and low wellbeing.

### Factors influencing wellbeing among high-achieving students

Student wellbeing is shaped by more than academic performance. It develops within linked systems including the individual, school, family, and broader sociocultural context, and influences often combine interactively rather than additively (Bronfenbrenner, 1979). For high achievers, this ecology can be especially consequential: strong ability and motivation often coexist with sustained academic pressure, perfectionistic expectations, and frequent social comparison, carrying psychological costs.

From a need-support perspective, wellbeing depends partly on whether personal resources and supportive relationships allow achievement to be experienced as meaningful and self-endorsed. Resilience and a growth mindset are positively related to subjective wellbeing and may help maintain positive affect and meaning under high demands (Zhao et al., 2024). At school, a safe, fair, supportive climate and a strong sense of belonging are consistently associated with better wellbeing (Obeidat et al., 2024), whereas bullying and low perceived safety relate to greater distress and lower life satisfaction (Xu and Fang, 2021). Family contexts also matter: warm, autonomy-supportive parenting and appropriately calibrated parental involvement are associated with higher life satisfaction, self-esteem, and emotional health in adolescence (Pérez-Fuentes et al., 2019; Wang and Sheikh-Khalil, 2014). Together, these findings suggest that high achievers are more likely to show a high achievement–high wellbeing pattern when belonging, support, and safety are present across school and family settings. When these resources are chronically limited, especially in high-pressure schools or high-expectation families, a high achievement–low wellbeing pattern becomes more likely (Luthar et al., 2020).

A cost-focused perspective highlights a complementary mechanism: the subjective costs required to sustain achievement. EVT emphasizes emotional burden, time demands, and opportunity costs in high-demand contexts. Persistently high anxiety, fatigue, and guilt can erode wellbeing even as performance remains strong (Eccles and Wigfield, 2020; Flake et al., 2015). Academic burnout research aligns with this view, showing that where high engagement co-occurs with high pressure, high achievers are more vulnerable to emotional exhaustion and cynicism and report lower wellbeing (Salmela-Aro and Upadyaya, 2020). Perfectionism and fear of failure may intensify these dynamics by tying self-worth to academic outcomes, supporting short-term performance while increasing longer-term psychological risk. Emotion regulation and coping also shape this pathway: cognitive reappraisal may reduce stress impacts on wellbeing, whereas rumination and avoidance may strengthen the link between pressure and low wellbeing.

Overall, wellbeing among high achievers reflects a balance between need-supportive resources and accumulating motivational costs. When support is strong and costs remain manageable, high achievement is more likely to coincide with positive affect and personal growth; when support is limited and costs build, high achievement is more likely to co-occur with emotional exhaustion and lower subjective wellbeing. Consistent with this framing, our model construction and variable selection prioritize indicators of school and family support, belonging and perceived safety, as well as academic anxiety, engagement intensity, and perceived pressure, to clarify systematic differences between high achievement–high wellbeing and high achievement–low wellbeing students.

### Applications of explainable machine learning in predicting achievement and wellbeing

Explainable machine learning comprises modeling and explanation techniques that help users understand how predictions are produced, supporting transparency and accountability in model-based decisions (Guidotti et al., 2018). In educational data mining, machine learning is widely used to predict academic performance, dropout risk, and online learning patterns. Compared with linear regression, ensemble methods such as random forests and XGBoost better capture non-linear relations and higher-order interactions in multi-source educational data and often achieve stronger predictive performance (Ahmed et al., 2025). However, these models are often difficult to interpret, limiting their value for instructional design and targeted support.

To bridge this gap, recent studies pair predictive models with explanation methods such as SHAP and LIME in grade prediction and learning analytics. Nachouki et al. (2023) used random forests and feature-importance analyses, showing that behavioral indicators such as classroom participation and assignment completion strongly contributed to course-grade prediction across courses. Zine et al. (2025) applied SHAP to predictors of online-learning readiness and found consistent positive contributions from self-regulated learning and access to technology. Such approaches shift attention from identifying at-risk students to clarifying which factors drive risk in a given setting.

Explainable machine learning has also been applied to student wellbeing and mental health. Using cross-national data, King et al. (2024) compared LightGBM with logistic regression and reported stable predictors of wellbeing across cultures, including school belonging, parental support, and meaning in learning. Related work applies SHAP to psychological distress, stress, and dropout risk, providing global summaries and individual-level explanations of model outputs (Zhai et al., 2025). Overall, this literature indicates that explainable machine learning can support accurate prediction while revealing feature patterns used by models to predict complex educational outcomes, informing more targeted intervention planning.

### Research gaps

Although extensive research has examined the relationship between academic achievement and student wellbeing, three gaps remain. Much of the literature focuses on the average association between achievement and wellbeing, with less attention to differences within the high-achieving group. This distinction matters because high achievement may coexist with either high or low wellbeing. As a result, the overall achievement-wellbeing association does not fully explain why some students remain psychologically well while others report low wellbeing despite similarly strong academic performance.

Existing studies have also identified important correlates of student wellbeing, including school belonging, family support, peer relationships, perceived safety, anxiety, and bullying. These factors, however, are often examined as separate predictors or additive correlates. This approach limits our understanding of whether hidden low wellbeing among high-achieving students reflects isolated deficits or revealing feature patterns used by models to predict complex educational outcomes and heightened cost-related pressures co-occur. The central issue, therefore, is not only which factors matter, but how they jointly differentiate HA-LW students from HA-HW students.

Machine-learning approaches are increasingly used in educational research, but many studies emphasize classification performance or global feature importance. Less attention has been given to how predictive signals can be interpreted through theory. In the context of high-achieving students, explainable machine learning may be especially useful if it moves beyond ranking predictors and helps clarify how support-related and cost-related signals are jointly organized in the prediction of hidden low wellbeing.

### The present study

This study examines hidden low wellbeing among high-achieving students using PISA 2022 data from 15 countries. Rather than treating academic achievement as sufficient evidence of positive adjustment, it focuses on students who reach relatively high levels of mathematics performance but differ markedly in subjective wellbeing. It compares high-achieving students with high wellbeing (HA-HW) and high-achieving students with low wellbeing (HA-LW), shifting the focus from the general association between achievement and wellbeing to differences within the high-achieving group.

Guided by Self-Determination Theory and Expectancy-Value Theory, the study examines hidden low wellbeing through a support-cost predictive-pattern perspective. Support-related signals include school belonging, family support, peer relationships, perceived safety, emotion regulation, and related contextual resources. Cost-related or risk-related signals include mathematics anxiety, bullying victimization, and self-directed learning difficulties. From this perspective, HA-LW status may not be explained by a single risk factor, but by the joint pattern of weakened supportive resources and increased costs among students who remain academically successful while reporting low wellbeing.

To examine this issue, the study uses explainable machine learning as a theory-guided analytic tool. The aim is not simply to maximize classification accuracy, but to identify which indicators most clearly distinguish HA-LW from HA-HW students and how these indicators are organized in the model's explanation. This approach links predictive evidence with theoretically interpretable support-related and cost-related signals.

The study addresses the following research questions:

RQ1: Which psychological, school, family, and learning-experience indicators best distinguish high-achieving students with low wellbeing from those with high wellbeing?

RQ2: Do the most informative indicators correspond to theoretically meaningful support-related and cost-related signals?

RQ3: How are these indicators jointly organized in the model's explanation of HA-LW and HA-HW profiles?

## Materials and methods

### Data and sample definition

This study used student-level data from the OECD Programme for International Student Assessment (PISA) 2022 (OECD, 2023, 2024). The analytic sample came from the 15 education systems that administered the wellbeing questionnaire (OPTION_WBQ = 1): the United Arab Emirates, Brazil, Costa Rica, Spain, France, Hong Kong (China), Hungary, Ireland, Macao (China), Mexico, the Netherlands, New Zealand, Panama, Saudi Arabia, and Slovenia. PISA 2022 assessed in-school students aged about 15 years, specifically between 15 years and 3 months and 16 years and 2 months. The student questionnaire covered socioeconomic background, school experiences, learning motivation, emotional experiences, and learning behaviors.

To identify academically successful students who reported low wellbeing, high achievement was defined within each education system rather than in the pooled international sample. Consistent with prior PISA-based machine learning research using within-system achievement criteria, students were classified as relatively high-achieving when their PV1MATH score fell within the top 25% of mathematics performance in their education system (Zheng et al., 2024). This criterion limits the influence of cross-national differences in overall achievement and identifies students performing well within their local educational context. Thus, high-achieving refers to relative high achievement within an education system, not to a narrowly defined gifted or elite-achievement group.

Subjective wellbeing was measured with the PISA life satisfaction item ST016Q01NA, which asks students to rate overall life satisfaction on an 11-point scale from 0 to 10. Life satisfaction is commonly used in PISA-based research as an indicator of students' subjective wellbeing (King et al., 2024; Li and Ho, 2024; OECD, 2017). Following OECD reporting conventions and prior threshold-based approaches, scores of 0 to 4 indicated low life satisfaction, whereas scores of 7 to 10 indicated high life satisfaction (OECD, 2017). Students scoring 5 or 6 were excluded to sharpen the contrast between the two wellbeing profiles. Among relatively high-achieving students, 6451 students with intermediate scores were excluded from the analytic comparison. The final comparison focused on relatively high-achieving students with high wellbeing (HA-HW) and relatively high-achieving students with low wellbeing (HA-LW), with wellbeing status defined by life satisfaction.

The original dataset contained 135,348 students from the 15 education systems that administered the wellbeing questionnaire. The analytic sample was restricted to students who met the within-system top-quartile criterion for mathematics performance, reported either low or high life satisfaction based on the thresholds above, and had valid responses on the required variables after preprocessing. The final analytic sample included 24,947 relatively high-achieving students, including 20,862 in the HA-HW group and 4,085 in the HA-LW group. Table 1 shows the analytic sample distribution across education systems.

**Table 1:** Analytic sample distribution across the 15 education systems.

PISA code	Education system	Total *n*	HA-HW *n*	HA-LW *n*	HA-LW %
ARE	United Arab Emirates	4,367	3,375	992	22.7
BRA	Brazil	1,746	1,411	335	19.2
CRI	Costa Rica	1,074	930	144	13.4
ESP	Spain	6,100	5,391	709	11.6
FRA	France	1,299	1,125	174	13.4
HKG	Hong Kong (China)	1,038	863	175	16.9
HUN	Hungary	1,208	1,075	133	11.0
IRL	Ireland	1,094	893	201	18.4
MAC	Macao (China)	824	656	168	20.4
MEX	Mexico	1,246	1,066	180	14.4
NLD	Netherlands	970	909	61	6.3
NZL	New Zealand	884	686	198	22.4
PAN	Panama	587	460	127	21.6
SAU	Saudi Arabia	1,263	1,075	188	14.9
SVN	Slovenia	1,247	947	300	24.1
**Total**		**24,947**	**20,862**	**4,085**	**16.4**

Because PISA uses a two-stage stratified cluster sampling design, the final student weight (W_FSTUWT) was included in the primary supervised learning analysis (OECD, 2024). In the GBM model, student weights were used as sample weights during fitting, and weighted AUC served as the main test-set performance indicator. Weighted threshold-dependent metrics were calculated as supplementary diagnostics, and unweighted AUC was reported as a robustness check. Although PISA provides 10 plausible values to account for uncertainty in achievement estimation, PV1MATH was used as the operational achievement indicator in the supervised learning pipeline (OECD, 2024; Wu, 2005). This choice allowed a single reproducible classification rule to be applied consistently across sample definition, model training, feature selection, and SHAP-based interpretation. Because the study aimed to predict differences between high-achieving students with contrasting wellbeing profiles rather than estimate population achievement parameters, PV1MATH was considered acceptable for the primary analysis.

## Measures

### Achievement-wellbeing profiles

The outcome variable distinguished two achievement-wellbeing profiles among relatively high-achieving students. Students with relatively high mathematics achievement and high life satisfaction were coded as HA-HW, whereas those with relatively high mathematics achievement and low life satisfaction were coded as HA-LW. In the machine learning models, HA-LW was the focal class because the study sought to identify patterns associated with low life satisfaction among students with relatively strong academic performance. The HA-LW category should therefore be read as a life-satisfaction-based profile, not as a clinical diagnosis or causal risk classification.

### Academic achievement

Academic achievement was measured using mathematics performance in PISA 2022. As noted above, students were classified as relatively high-achieving when their PV1MATH score placed them in the top quartile of mathematics performance within their education system (OECD, 2024; Zheng et al., 2024). This within-system criterion was used because absolute mathematics performance varies across countries and economies. It identified students who performed well relative to their own educational context.

### Subjective wellbeing: life satisfaction

Subjective wellbeing was measured with the PISA life satisfaction item ST016Q01NA, which asks students to rate overall life satisfaction on an 11-point scale from 0 to 10. Following OECD reporting conventions and prior PISA-based studies of student wellbeing, scores from 0 to 4 indicated low life satisfaction, whereas scores from 7 to 10 indicated high life satisfaction (King et al., 2024; Li and Ho, 2024; OECD, 2017). This threshold-based grouping created a clearer contrast between relatively high-achieving students with low and high life satisfaction. Because the measure consists of a single life satisfaction item, the outcome should be interpreted as a life-satisfaction-based indicator of wellbeing status rather than as a comprehensive measure of psychological, emotional, or social wellbeing.

### Predictor variables

Predictors were drawn from the PISA 2022 student questionnaire and covered psychological, school, family, and learning-experience indicators (OECD, 2023, 2024). The initial set included 98 student-level characteristics. Variables with more than 30% missingness were removed during preprocessing, leaving 67 features for model development. These variables were not selected to impose a predetermined theoretical structure on the model. Instead, they provided a broad pool of candidate indicators from which the machine learning models could identify prediction-relevant information.

For interpretation, the retained predictors were grouped into support-related signals, cost-related or risk-related signals, and background or supplementary signals. Support-related signals included school belonging, family support, perceived safety, relationship quality at school, emotion regulation, creative family environment, and self-directed learning efficacy. These variables were interpreted as relational, emotional, contextual, or regulatory resources relevant to life satisfaction among high-performing students. Cost-related or risk-related signals included mathematics anxiety, bullying victimization, and self-directed learning difficulties. These variables were interpreted as psychological, social, or regulatory burdens that may distinguish relatively high-achieving students with low life satisfaction from those with high life satisfaction. Gender and openness to art or creative engagement were retained as background or supplementary signals when selected by the model.

Table 2 provides plain-language descriptions of the PISA variables retained in the final 12-feature model, improving readability for readers less familiar with PISA variable codes.

**Table 2:** Plain-language descriptions of the final 12 predictors retained in the GBM Model.

Variable code	Plain-language label	Interpretation category	Description in this study
BELONG	School belonging	Support-related signal	Students' perceived sense of acceptance and connection at school.
EMOCOAGR	Emotion regulation	Support-related signal	Students' perceived ability to regulate emotional responses.
FAMSUP	Family support	Support-related signal	Supportive resources perceived from family.
FEELSAFE	Perceived safety	Support-related signal	Students' perceived safety in the school environment.
RELATST	Relationship quality at school	Support-related signal	Quality of students' school-based relationships.
CREATFAM	Creative family environment	Support-related signal	Family environment supportive of creative engagement or expression.
SDLEFF	Self-directed learning efficacy	Support-related signal	Students' confidence in managing self-directed learning.
PROBSELF	Self-directed learning difficulties	Cost-related/risk-related signal	Difficulties in managing self-directed learning.
BULLIED	Bullying victimization	Cost-related/risk-related signal	Exposure to bullying or peer victimization.
ANXMAT	Mathematics anxiety	Cost-related/risk-related signal	Anxiety or worry related to mathematics learning.
gender	Gender	Background signal	Student gender.
OPENART	Openness to art/creative engagement	Background or supplementary signal	Openness toward art-related or creative activities.

This theoretical classification was used to guide interpretation of the explainable machine-learning results. It did not impose restrictions on model estimation. Instead, it provided a framework for interpreting whether the most informative predictors could be interpreted as support-related and cost-related predictive signals.

## Analytical procedure

### Data preparation and preprocessing

The analytic sample was limited to students from the 15 PISA 2022 education systems that administered the wellbeing questionnaire (OPTION_WBQ = 1) (OECD, 2023, 2024). Within each education system, students were classified as relatively high-achieving when their PV1MATH score was at or above the 75th percentile. Life satisfaction was measured using ST016Q01NA. Scores of 0 to 4 indicated low life satisfaction, whereas scores of 7 to 10 indicated high life satisfaction. Students scoring 5 or 6 were excluded from the main comparison to create a clearer contrast between the two profiles (OECD, 2017; King et al., 2024; Li and Ho, 2024).

The initial predictor pool comprised 98 student-level variables from the PISA 2022 student questionnaire. Variables with more than 30% missingness were removed, leaving 67 candidate predictors. For the retained variables, missing values were handled using k-nearest-neighbor donor identification and hot-deck imputation, with casewise deletion applied when required (Alvarez-Garcia et al., 2024a,b). Outliers were treated using a quartile-based rule.

The final student weight (W_FSTUWT) was used for weighted model fitting and evaluation but was not included as a predictor. Education system identifiers, sampling weights, and outcome variables were removed from the predictor matrix to avoid information leakage.

### Model development and comparison

The analytic sample was divided into a 70% training set and a 30% validation set, stratified by achievement-wellbeing profile, with HA-LW treated as the focal class. Eleven classifiers were compared: AdaBoost, artificial neural network, decision tree, CatBoost, Gradient Boosting Machine, k-nearest neighbors, LightGBM, logistic regression, random forest, support vector machine, and XGBoost.

Model tuning was conducted within the training set using stratified K-fold cross-validation, grid search, and targeted manual refinement. W_FSTUWT was supplied during model fitting when the algorithm supported sample weights. Model performance was evaluated on the validation set.

Weighted AUC was used as the primary discrimination metric, and unweighted AUC was reported as a robustness check. Because HA-LW students were fewer than HA-HW students, threshold-dependent metrics were also reported for the HA-LW focal class, including precision, recall, F1 score, and balanced accuracy.

### Feature selection and model interpretation

SHAP-assisted feature selection was used to obtain a parsimonious model for interpretation (Lundberg and Lee, 2017). Predictors were first ranked in the training set by weighted mean absolute SHAP values. Candidate models using different numbers of top-ranked features were then trained and evaluated on the validation set. The final feature set was chosen by balancing discrimination and interpretability; when a smaller feature set achieved a weighted AUC comparable to that of larger feature sets, the smaller set was preferred.

AUC differences between candidate GBM models were evaluated using bootstrap tests. The selected GBM model was then interpreted with SHAP values. Global SHAP importance summarized each feature's average contribution to model discrimination between HA-LW and HA-HW students. In SHAP summary plots, positive SHAP values shifted predictions toward HA-LW, whereas negative values shifted predictions toward HA-HW. Point color indicated the observed feature value.

**Figure 1:**
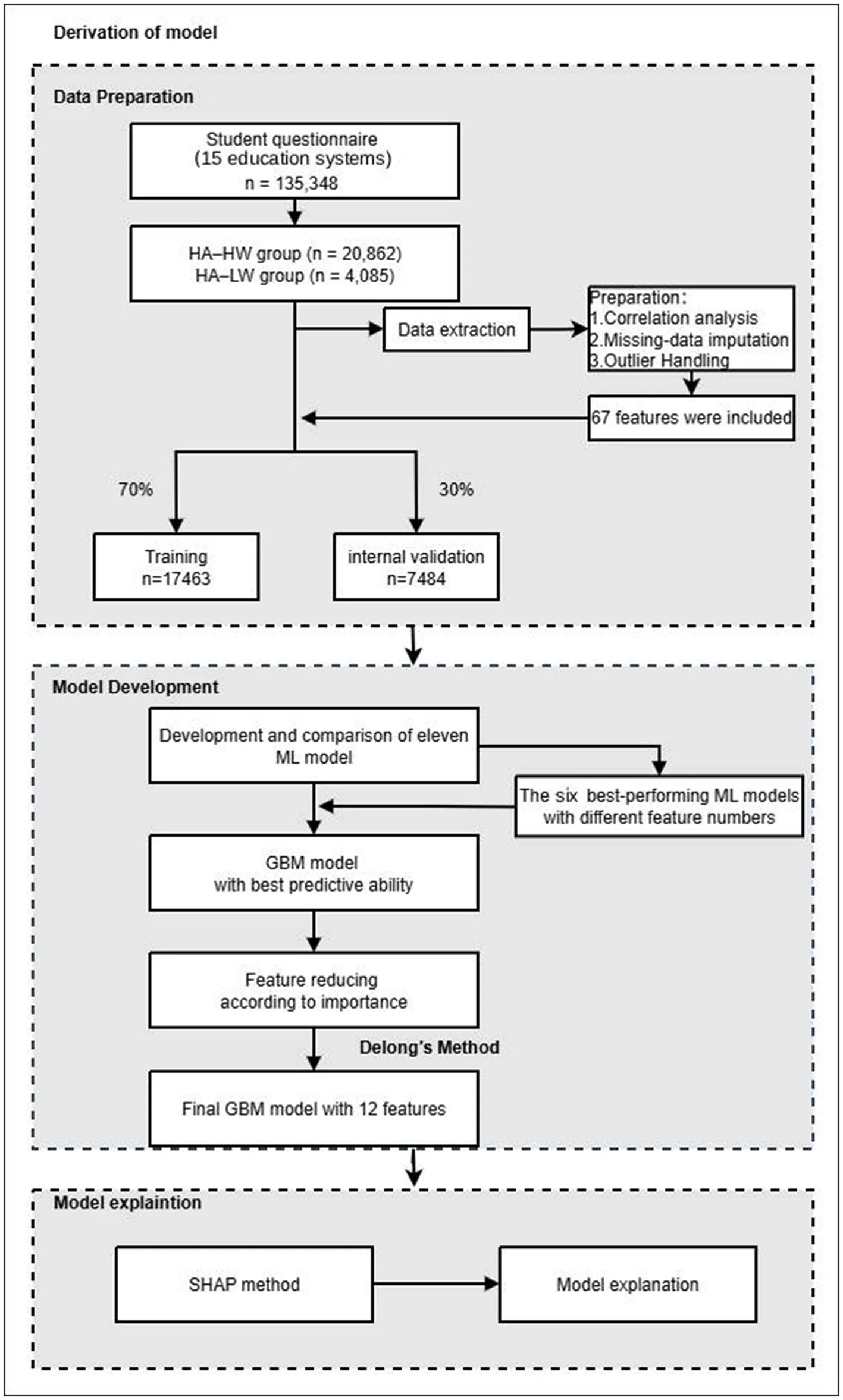
Process of data analysis.

SHAP interaction values summarized pairwise interaction contributions within the final model's prediction function. These interaction results were interpreted strictly as model-based explanations, not as causal effects, psychological networks, or evidence of latent student configurations. Figure 1 shows the full analytic workflow.

## Results

### Model performance and selection

**Figure 2:**
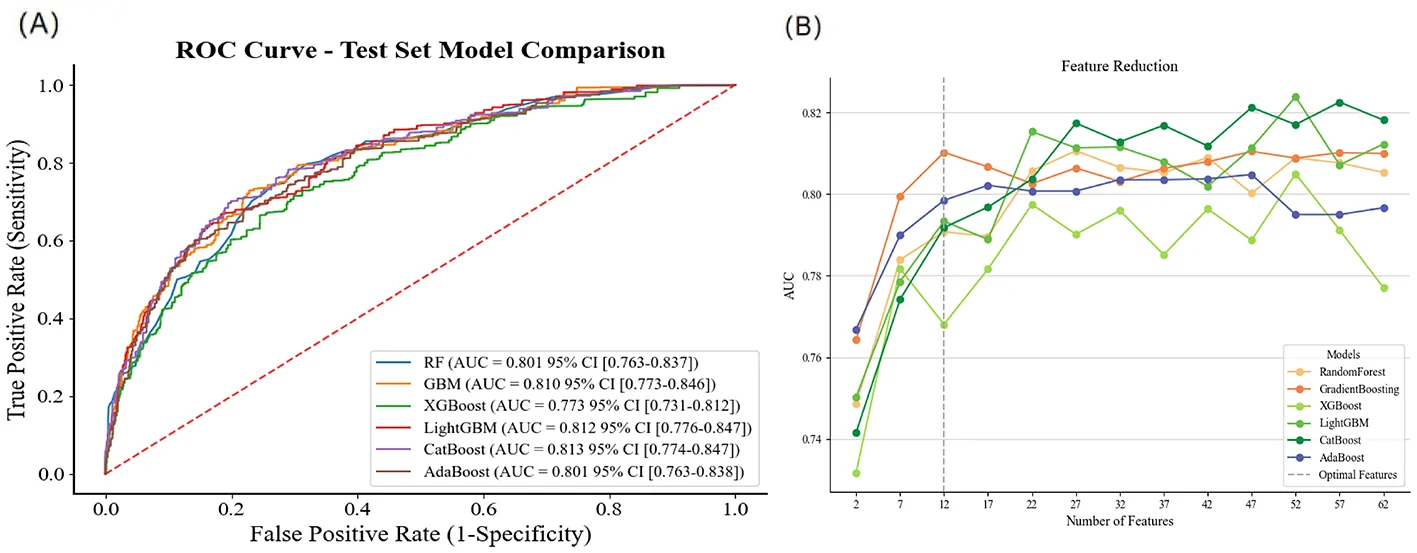
Model performance comparison. **(A)** ROC curves of the top six machine-learning models. **(B)** AUC values of the top six models across different feature-set sizes.

We first identified a model that could distinguish relatively high-achieving students with low life satisfaction (HA-LW) from those with high life satisfaction (HA-HW) and provide sufficient stability for later interpretation. Using the same 70/30 stratified training-validation split and weighted evaluation procedure, 11 machine learning classifiers were compared, with weighted AUC as the primary performance metric (Figure 2A). CatBoost produced the highest weighted AUC (0.813), followed closely by LightGBM (0.812) and GBM (0.810). These three models ranked at the top, suggesting that boosting-based methods were well suited to distinguishing life-satisfaction profiles within the relatively high-achieving group.

Although CatBoost and LightGBM yielded slightly higher weighted AUC values, GBM showed smaller fluctuations across feature-set sizes (Figure 2B). This stability was important because the study aimed not only to compare model performance but also to identify and interpret prediction-relevant signals associated with hidden low wellbeing among relatively high-achieving students. GBM was therefore retained as the primary model for feature reduction and SHAP-based explanation.

### Selection of a parsimonious feature set

The next step was to identify a feature set that maintained discrimination while improving interpretability. GBM-based feature reduction was conducted using SHAP-assisted ranking. Candidate models with different numbers of top-ranked features were compared to assess whether a smaller feature set could achieve discrimination comparable to that of larger models.

**Figure 3:**
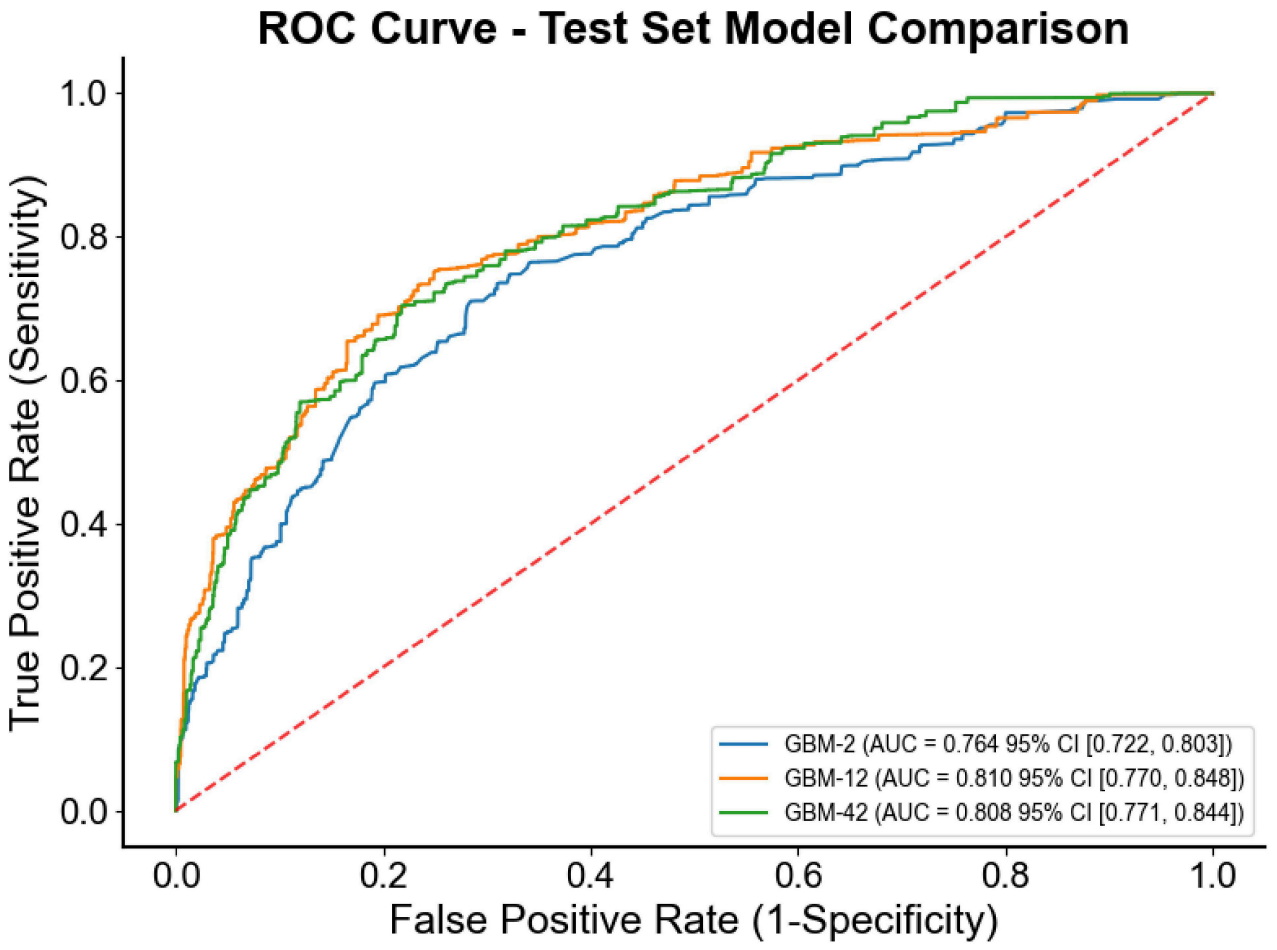
ROC curves of GBM models with different feature counts.

The 12-feature model offered the best balance between discrimination and interpretability (Figure 3). Weighted bootstrap tests for AUC differences showed that the 12-feature model performed substantially better than the 2-feature model in identifying HA-LW students, whereas the 42-feature model did not meaningfully improve on the 12-feature model. Increasing the feature set from 12 to 42 therefore did not materially improve discrimination. The 12-feature model retained the main predictive information while allowing a more focused interpretation of the signals distinguishing HA-LW students from HA-HW students.

**Figure 4:**
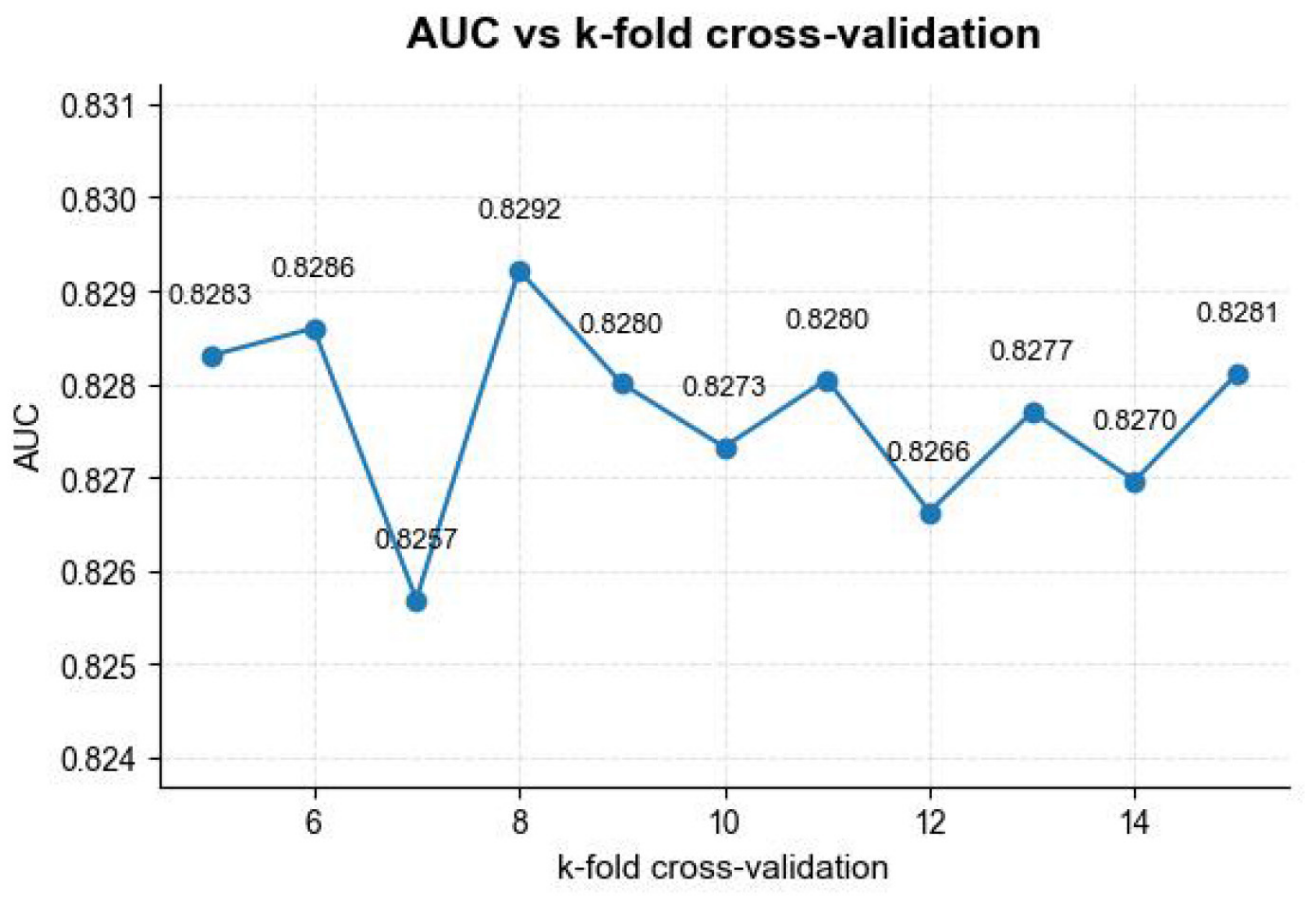
Stability of the selected 12-feature GBM model across K-fold specifications.

To examine the stability of the selected feature set, stratified K-fold cross-validation was conducted with K varying from 5 to 15 (Figure 4). Model performance remained stable across fold specifications, with the highest mean AUC observed at K = 8 (AUC = 0.8292). These findings indicate that the 12-feature GBM model retained sufficient discriminative performance while providing a compact basis for interpretation.

The final 12-feature set included BELONG, EMOCOAGR, FAMSUP, FEELSAFE, RELATST, CREATFAM, PROBSELF, BULLIED, ANXMAT, SDLEFF, gender, and OPENART. These features were used in the subsequent SHAP analyses to examine which signals contributed most strongly to discrimination between HA-LW and HA-HW students and how the model combined these signals in its explanations.

Because HA-LW was the minority class, we further examined threshold-dependent metrics for the final 12-feature GBM model as supplementary diagnostics. At the default 0.50 threshold, the model showed relatively high weighted precision but low weighted recall, indicating a conservative classification pattern for HA-LW students. Specifically, weighted precision was 0.706, weighted recall was 0.276, and weighted F1 was approximately 0.396. When the Youden-index threshold was used, weighted recall increased to 0.753 and weighted balanced accuracy increased to approximately 0.751, but weighted precision decreased to 0.364 and weighted F1 was approximately 0.491. This threshold trade-off is expected given the class imbalance and indicates that the model should be interpreted as supporting group-level discrimination and explanation rather than individual-level screening. The weighted PR-AUC was 0.534, with an unweighted PR-AUC of 0.556, providing an additional performance summary under class imbalance. Full threshold-dependent metrics are reported in Supplementary Table 2.

### Global SHAP explanation of support-cost signals

**Figure 5:**
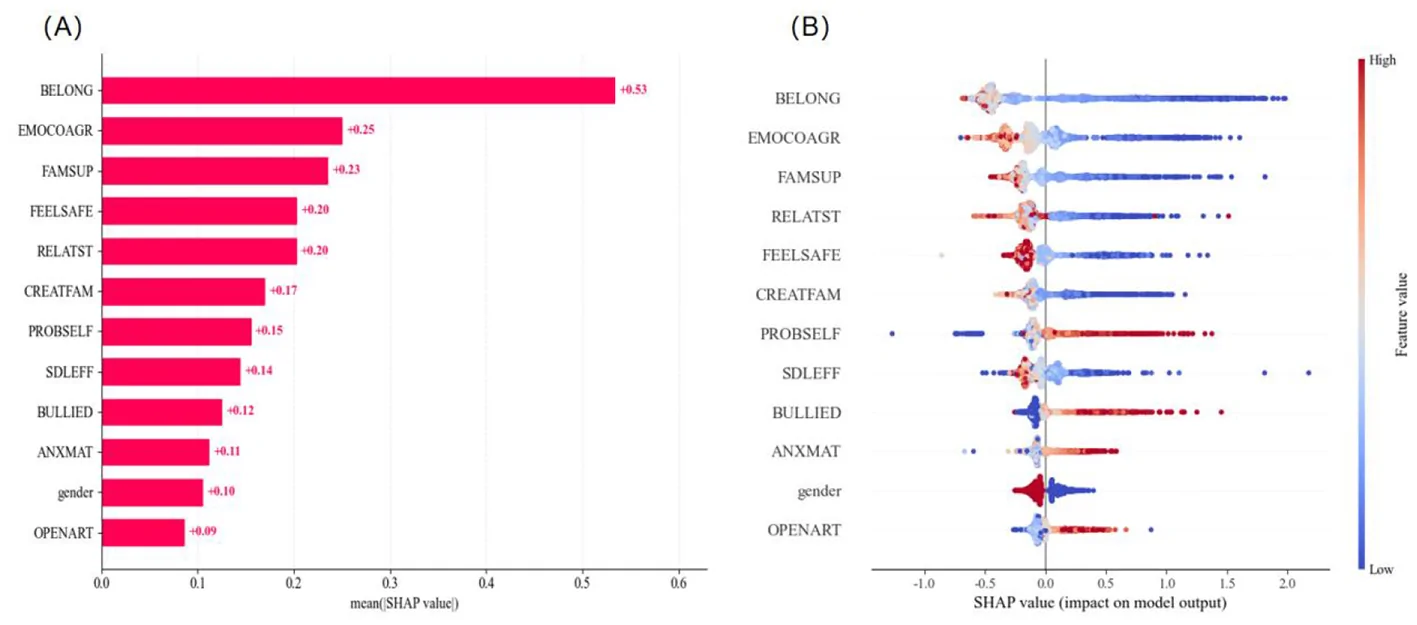
Global model explanation using SHAP. **(A)** SHAP summary bar plot. **(B)** SHAP summary dot plot.

SHAP analysis was used to interpret the final 12-feature GBM model. Weighted mean absolute SHAP values indicated that BELONG contributed most strongly to the model's discrimination between HA-LW and HA-HW students (0.53), followed by EMOCOAGR (0.25) and FAMSUP (0.23) (Figure 5A). Other important features included FEELSAFE (0.20), RELATST (0.20), CREATFAM (0.17), PROBSELF (0.15), SDLEFF (0.14), BULLIED (0.12), and ANXMAT (0.11). Gender (0.10) and OPENART (0.09) were retained in the final model, but their contributions were smaller than those of the core psychological, school, family, and learning-experience indicators.

These findings address RQ1 by showing that, among relatively high-achieving students, school belonging was the strongest model-based signal differentiating HA-LW from HA-HW students. It was followed by emotion regulation, family support, perceived safety, relationship quality at school, creative family environment, self-directed learning difficulties, self-directed learning efficacy, bullying victimization, and mathematics anxiety. The model therefore distinguished HA-LW and HA-HW students mainly through relational, emotional, contextual, and learning-experience signals, rather than through background variables alone.

The SHAP summary plot further clarified the direction of these contributions (Figure 5B). Lower BELONG values were more often associated with positive SHAP values, indicating that low school belonging increased the model's predicted probability of HA-LW status. Higher BELONG values were more often associated with negative SHAP values, indicating that stronger school belonging shifted predictions toward HA-HW. EMOCOAGR showed a similar pattern: lower emotion regulation increased the predicted probability of HA-LW status, whereas higher emotion regulation shifted predictions toward HA-HW. FAMSUP, RELATST, and FEELSAFE also showed support-oriented predictive directions, with higher values generally shifting predictions away from HA-LW.

CREATFAM and SDLEFF followed the same support-oriented pattern. Lower levels of creative family environment were more often associated with HA-LW prediction, whereas higher levels tended to shift predictions toward HA-HW. Similarly, higher self-directed learning efficacy more often contributed negatively to HA-LW prediction, suggesting that learning efficacy functioned as a regulatory resource in the model's discrimination between the two profiles.

PROBSELF, BULLIED, and ANXMAT showed cost-oriented or risk-oriented predictive contributions. Higher self-directed learning difficulties shifted predictions toward HA-LW. Higher bullying victimization and mathematics anxiety also tended to contribute positively to HA-LW prediction. These patterns address RQ2 by showing that the most informative indicators corresponded to theoretically meaningful support-related and cost-related signals in the model. BELONG, EMOCOAGR, FAMSUP, FEELSAFE, RELATST, CREATFAM, and SDLEFF functioned mainly as support-related predictive signals, whereas PROBSELF, BULLIED, and ANXMAT functioned mainly as cost-related or risk-related predictive signals. Gender and OPENART were best interpreted as background or supplementary signals rather than central sources of model discrimination.

### Model-based interaction structure of support-cost signals

SHAP interaction values were used to examine whether HA-LW and HA-HW profiles differed not only in individual feature importance but also in how the model combined predictors. In the resulting interaction networks, nodes represented features, and edges represented pairwise interaction contributions within the model's prediction function. These networks were interpreted strictly as predictive interaction structures, not as causal networks, psychological mechanisms, or latent student configurations.

**Figure 6:**
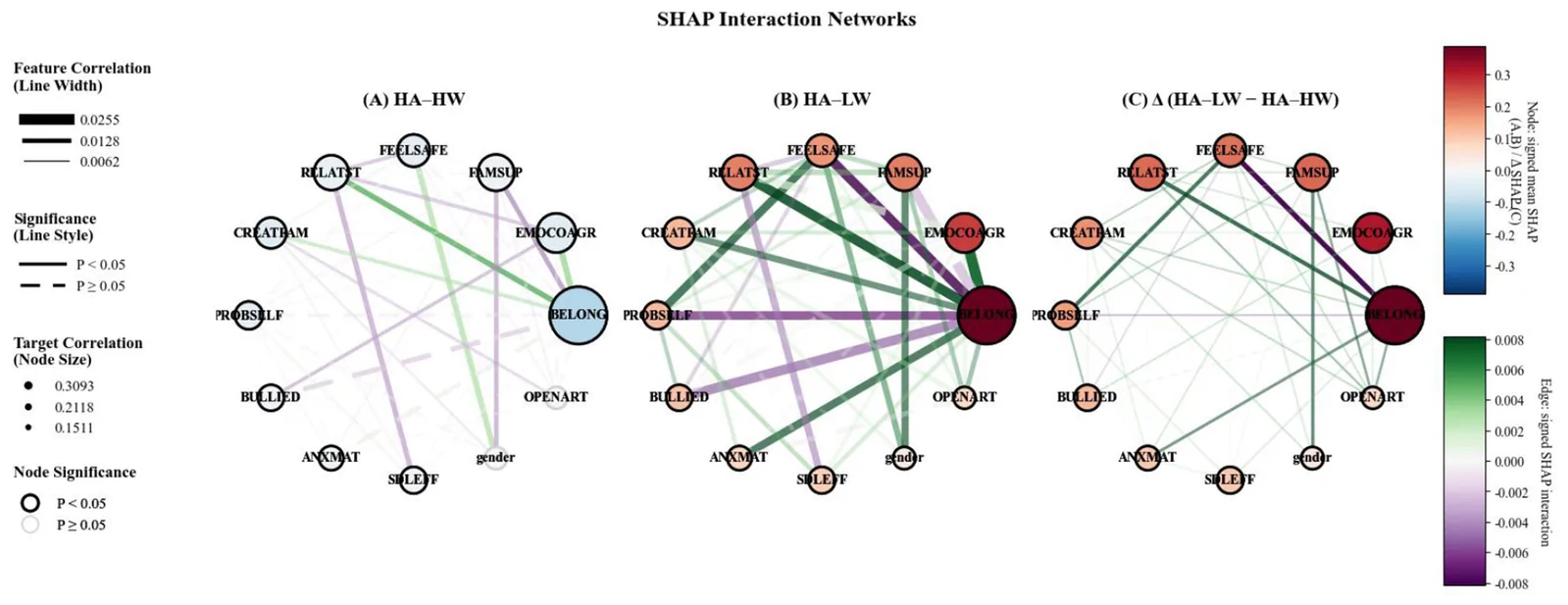
Model-based SHAP interaction networks for support-cost signals. **(A)** Interaction structure for HA-HW students. **(B)** Interaction structure for HA-LW students. **(C)** Between-group differences in SHAP interaction structure.

Figure 6A shows the interaction structure for the HA-HW group. The HA-HW network was relatively dispersed. Although several links appeared among core features, the model's explanation of HA-HW status was not strongly centered on a single dominant node. This pattern suggests that the model classified HA-HW students through a more distributed set of support-related signals.

Figure 6B shows a different pattern for the HA-LW group. Compared with HA-HW classification, the model's explanation of HA-LW status was more concentrated around BELONG and its links with other key signals. Among the top ten edges with the largest group differences, six involved BELONG: BELONG-EMOCOAGR, BELONG-BULLIED, BELONG-RELATST, BELONG-FAMSUP, BELONG-CREATFAM, and BELONG-PROBSELF. The largest difference appeared for BELONG-EMOCOAGR, where absolute interaction strength increased from 0.0134 in HA-HW to 0.0272 in HA-LW.

These results indicate that BELONG was not only the strongest individual predictor but also a central feature in the model's interaction-based explanation of HA-LW classification. BELONG-centered interactions linked support-related signals, including emotion regulation, relationship quality at school, family support, and creative family environment, with cost-related or risk-related signals, including bullying victimization and self-directed learning difficulties. Additional high-difference edges, such as EMOCOAGR-RELATST and EMOCOAGR-BULLIED, further suggest that the model used combinations of emotional, relational, and risk-related features to distinguish HA-LW students.

Figure 6C shows that group differences were more pronounced in the interaction structure than in overall shifts in feature importance. The clearest differences involved stronger model-based coupling between belonging and relationship-related or safety-related signals, together with tighter links between support-related and cost-related predictors. These findings address RQ3. The model's explanation suggests that HA-LW classification relied more strongly on BELONG-centered combinations of support-related and cost-related signals, whereas HA-HW classification relied more on a dispersed pattern of support-related resources.

Taken together, the SHAP interaction results suggest that the model did not distinguish HA-LW students through a single elevated risk factor. Instead, discrimination depended on combinations of support-related and cost-related predictors, especially those centered on school belonging. This interpretation remains predictive rather than causal, but it clarifies how the final GBM model organized the key signals differentiating HA-LW from HA-HW students.

## Discussion

This study used explainable machine learning to examine hidden low wellbeing among relatively high-achieving students in a cross-national PISA 2022 sample. The aim was not to develop an individual diagnostic tool, but to identify model-based signals that distinguished relatively high-achieving students with low life satisfaction (HA-LW) from those with high life satisfaction (HA-HW). The results showed that a compact 12-feature GBM model retained discriminative performance comparable to larger feature sets and remained stable across K-fold specifications. SHAP analyses identified school belonging (BELONG) as the strongest differentiating feature. Emotion regulation, family support, relationship quality at school, perceived safety, creative family environment, and self-directed learning efficacy generally shifted predictions toward HA-HW, whereas mathematics anxiety, bullying victimization, and self-directed learning difficulties shifted predictions toward HA-LW. SHAP interaction analyses further suggested that HA-LW classification relied more strongly on BELONG-centered combinations of support-related and cost-related signals than did HA-HW classification.

These findings should be read as predictive, model-based evidence. They do not show that school belonging or other predictors causally produce low wellbeing, nor do they establish a latent psychological configuration among students. Instead, they indicate that, within the final GBM model, hidden low wellbeing among relatively high-achieving students was best distinguished by a combination of relational, emotional, family, peer, and learning-experience signals. This interpretation is consistent with an integrated Self-Determination Theory and Expectancy-Value Theory framework, which emphasizes both supportive resources and subjective costs in students' academic and wellbeing experiences.

### School belonging in the model's explanation of HA-LW classification

The strongest and most consistent model-based finding concerned school belonging. BELONG had the largest weighted mean absolute SHAP value and showed the clearest directional pattern: lower belonging increased the model's predicted probability of HA-LW status, whereas higher belonging shifted predictions toward HA-HW. This finding is consistent with prior research linking school belonging to adolescent mental health and wellbeing (Arslan, 2021; Allen et al., 2024). The present study extends this evidence by showing that, even among relatively high-achieving students, school belonging remained the most informative signal for distinguishing low from high life satisfaction.

The SHAP interaction network further showed that several of the largest group differences involved BELONG. In the model's explanation of HA-LW classification, BELONG had stronger interaction contributions with emotion regulation, bullying victimization, relationship quality at school, family support, creative family environment, and self-directed learning difficulties. The largest difference appeared for the BELONG-EMOCOAGR edge, where absolute interaction strength was higher in the HA-LW explanation than in the HA-HW explanation. This pattern indicates that the model did not rely on lower belonging as an isolated feature. Instead, it combined belonging with emotional, relational, family, and learning-related signals when distinguishing HA-LW students.

This finding is theoretically meaningful but should not be interpreted causally. From the perspective of Self-Determination Theory, relatedness is a basic psychological need closely tied to wellbeing and adaptive functioning (Ryan and Deci, 2000, 2020). The present results are consistent with this view, but they do not show that low belonging reorganizes students' psychological systems. They show that belonging was central to the GBM model's prediction function. In practical terms, school belonging may serve as a useful monitoring signal for identifying groups of high-achieving students whose strong performance may coexist with low life satisfaction.

### Support-cost predictive patterns and the SDT-EVT interpretation

The second key finding was that the most informative model signals aligned with the support-cost perspective drawn from Self-Determination Theory and Expectancy-Value Theory. Higher values on support-related indicators, including BELONG, EMOCOAGR, FAMSUP, FEELSAFE, RELATST, CREATFAM, and SDLEFF, generally shifted predictions toward HA-HW. In contrast, higher values on cost-related or risk-related indicators, including PROBSELF, BULLIED, and ANXMAT, generally shifted predictions toward HA-LW. This pattern suggests that HA-LW and HA-HW students were not differentiated by achievement level itself, since both groups were relatively high achieving, but by the support-related and cost-related signals surrounding their academic profile.

Self-Determination Theory helps explain the support-related side of this pattern. Belonging, family support, relationship quality at school, perceived safety, and emotion regulation can be viewed as relational and regulatory resources that are theoretically relevant to wellbeing. In the present model, higher levels of these indicators tended to shift predictions toward HA-HW. This does not show that these resources cause high wellbeing, but it does indicate that the model used them to distinguish high-achieving students with high life satisfaction from those with low life satisfaction.

Expectancy-Value Theory helps explain the cost-related side of the pattern. Mathematics anxiety, self-directed learning difficulties, and bullying victimization can be viewed as psychological, regulatory, and social burdens that may accompany academic performance. In the present model, higher values on these indicators tended to shift predictions toward HA-LW. This finding matters because strong academic performance can mask such burdens. Students who continue to perform well may not be recognized as needing support if schools rely mainly on achievement indicators.

The interaction results further suggest that support-related and cost-related signals should not be interpreted only as separate predictors. In the HA-LW explanation, belonging was more strongly linked with both supportive resources and risk-related pressures. This does not establish a causal support-cost mechanism, but it shows that the model's discrimination depended on combinations of support-related and cost-related features. From an SDT-EVT perspective, this pattern is meaningful because high achievement may coexist with low life satisfaction when supportive resources are weaker and subjective costs are more pronounced.

### Contribution to research on high-achieving students

This study contributes to research on high-achieving students by shifting attention from achievement level alone to achievement-wellbeing profiles. Much of the existing literature has examined whether achievement and wellbeing are associated on average. The present findings show why that approach is limited. Even among students who were similarly high achieving within their education systems, life satisfaction profiles varied substantially, and these differences were reflected in relational, emotional, family, peer, and learning-experience signals.

This point is important because high-achieving students are often treated as a lower priority for support. When students perform well academically, teachers and schools may assume that they are coping effectively. The present findings challenge that assumption. HA-LW students were not characterized by low achievement, but by model-based signals that may be less visible in routine academic monitoring, including lower school belonging, lower emotion regulation, higher mathematics anxiety, bullying victimization, and self-directed learning difficulties. These indicators suggest that strong academic performance can coexist with low life satisfaction.

The study also extends machine learning research on student wellbeing. Previous cross-national work has identified robust predictors of student wellbeing, including school belonging and parental support (King et al., 2024). The present study builds on this work by focusing specifically on relatively high-achieving students and by examining how predictors contributed to model explanations. Its contribution is not only that BELONG, FAMSUP, or ANXMAT were important, but that these signals jointly helped the model distinguish hidden low wellbeing among students whose academic performance might otherwise suggest positive adjustment.

### Methodological implications of explainable machine learning

This study also has methodological implications. Explainable machine learning was useful because it linked predictive performance with interpretable model evidence. The 12-feature GBM model provided a compact representation of the main signals distinguishing HA-LW students from HA-HW students. SHAP values showed which features contributed most strongly to the model's predictions and how different feature values shifted predictions toward HA-LW or HA-HW. SHAP interaction values further offered a model-based way to examine whether prediction depended on isolated features or on combinations of features.

This does not mean that explainable machine learning replaces theory. The SHAP results were interpretable because they were examined through an SDT-EVT framework. Without that framework, the results would remain largely a ranked list of predictors and interactions. By organizing features into support-related and cost-related signals, the study translated model explanations into a theoretically meaningful account of how the GBM model distinguished HA-LW students from HA-HW students.

At the same time, the value of SHAP depends on careful interpretation. SHAP values describe contributions to model predictions, not causal effects. SHAP interaction values describe pairwise interaction contributions within the model's prediction function, not actual psychological networks among students. The study should therefore be understood as identifying predictive and explanatory patterns that can guide future theory testing, rather than as providing causal evidence about mechanisms or intervention effects.

## Practical implications

The findings have practical implications for schools and student-support systems. Academic performance alone should not be treated as evidence of positive adjustment. Relatively high-achieving students may still report low life satisfaction, especially when school belonging is low and cost-related pressures are high. Schools should therefore include wellbeing indicators in broad student-monitoring systems, including for students who perform well academically.

School belonging and perceived safety may be especially useful indicators in high-achievement contexts. Because BELONG was the strongest individual signal and was central to the model's interaction-based explanation of HA-LW classification, schools should monitor whether high-achieving students feel accepted, connected, and valued in the school community. Anti-bullying practices, fair teacher-student interactions, peer inclusion, and emotionally safe classrooms may help support wellbeing among academically successful students.

Support services should address both resources and costs. Reducing anxiety or bullying may be insufficient if students continue to experience weak belonging, limited family support, poor peer relationships, or low emotion regulation. Similarly, efforts to strengthen belonging may be less effective if students continue to experience high mathematics anxiety, self-directed learning difficulties, or adverse peer experiences. A coordinated approach is therefore more appropriate than a single-domain response.

These implications should be interpreted cautiously. The present model should not be used as a clinical or diagnostic screening tool for individual students. Instead, the findings can help schools identify broad areas for monitoring and support. For high-achieving students, the goal should not be to lower achievement expectations, but to ensure that achievement is not sustained at excessive psychological, social, or regulatory cost.

## Limitations and future research

Several limitations warrant consideration. First, HA-LW and HA-HW were defined through threshold-based grouping. This approach created a clear contrast between low and high life satisfaction profiles, but it also reduced information from the continuous distribution of life satisfaction. Students with intermediate scores of 5 or 6 were excluded from the main comparison. Although this exclusion sharpened the contrast between the two profiles, it may also have amplified group differences. Future studies should examine whether the findings remain stable when life satisfaction is modeled continuously, when intermediate scores are retained, or when alternative thresholds are applied.

Second, high achievement was defined as the top 25% of mathematics performance within each education system. This within-system criterion is useful for identifying students who are relatively high achieving in their own educational contexts, and it follows prior PISA-based research using relative achievement definitions. However, the top-quartile group should not be interpreted as a narrowly defined gifted or elite-achievement group. Some students in this group may be moderately, rather than exceptionally, high performing. Future research should test whether the same predictive patterns hold under stricter thresholds, such as the top 10% or top 15%.

Third, academic achievement was operationalized using PV1MATH. This decision allowed a single reproducible classification rule to be applied across sample definition, model training, feature selection, and SHAP interpretation. However, PISA provides multiple plausible values to represent uncertainty in achievement estimation. The present analysis did not test whether group assignment and model results were stable across all plausible values. Future studies should conduct sensitivity analyses using PV1MATH through PV10MATH.

Fourth, subjective wellbeing was measured with a single PISA life satisfaction item. Although life satisfaction is widely used in PISA-based research as an indicator of subjective wellbeing, it does not capture the multidimensional nature of wellbeing. Broader measures of emotional wellbeing, psychological functioning, school wellbeing, social relationships, and mental health may reveal additional patterns. Future research should examine whether the support-related and cost-related signals identified here remain stable when more comprehensive wellbeing measures are available.

Fifth, the analysis pooled students from 15 education systems. This approach provided a large cross-national sample and allowed the model to identify general predictive signals. However, school belonging, family support, bullying, safety, and life satisfaction may operate differently across cultural and educational contexts. The proportion of HA-LW students also varied across education systems. Future studies should examine country-specific models, leave-one-country-out validation, and multilevel machine learning approaches to test whether the main patterns generalize across contexts.

Sixth, the study used cross-sectional PISA 2022 data. The findings identify prediction-relevant signals and model-based interaction patterns, but they cannot establish causal pathways. The results do not show that low belonging, anxiety, bullying, or self-directed learning difficulties cause low wellbeing. Longitudinal, quasi-experimental, and intervention studies are needed to test temporal ordering and causal mechanisms.

Finally, SHAP and SHAP interaction analyses should be interpreted as tools for explaining model behavior. SHAP values describe contributions to model predictions, not causal effects. SHAP interaction values describe pairwise contributions within the model's prediction function, not psychological networks or latent configurations among students. Because HA-LW students constituted the minority class, threshold-dependent metrics should also be interpreted as supplementary diagnostics rather than evidence that the model is ready for individual-level screening. The present model is best understood as a tool for identifying predictive patterns and informing future theory testing, not as a diagnostic instrument.

## Data Availability

The original contributions presented in the study are included in the article/Supplementary material, further inquiries can be directed to the corresponding author.
