# Supplementary material for: Hidden low wellbeing among high-achieving students: model-based support-cost patterns revealed by explainable machine learning
Source: Front Psychol. 2026 Aug 25;17:1891921. doi: 10.3389/fpsyg.2026.1891921 (PMC13552010; doi:10.3389/fpsyg.2026.1891921)
Supplement: Supplementary file 1 [file Supplementary_file_1.docx]

Fig.S1 hot-deck imputation

Fig.S2 Distribution of Missing Values

Fig.S3 Comparison of Variable Distributions Before and After Hot-deck Imputation

TableS1 Feature

| Table Variables used in the case study | | |
| --- | --- | --- |
| **variable name** | **Type** | **Description** |
| BELONG | Num | Sense of belonging (WLE) |
| EMOCOAGR | Num | Emotional control (agreement) (WLE) |
| FAMSUP | Num | Family support (WLE) |
| FEELSAFE | Num | Feeling safe (WLE) |
| RELATST | Num | Quality of student-teacher relationships (WLE) |
| CREATFAM | Num | Creative peers and family environment (WLE) |
| PROBSELF | Num | Problems with self-directed learning (WLE) |
| BULLIED | Num | Being bullied (WLE) |
| ANXMAT | Num | Mathematics Anxiety (WLE) |
| SDLEFF | Num | Self-directed learning self-efficacy (WLE) |
| gender（ST004D01T） | Cat | Student Gender |
| OPENART | Num | Openness to Art and Reflection (WLE) |

Table S2. Threshold-dependent Performance Metrics for the Final 12-feature GBM Model

| Threshold rule | Threshold | Weighted AUC | Weighted PR-AUC | Weighted precision | Weighted recall | Weighted specificity | Weighted F1 | Weighted balanced accuracy | Interpretation |
| --- | --- | --- | --- | --- | --- | --- | --- | --- | --- |
| Default | 0.500 | 0.810 | 0.534 | 0.706 | 0.276 | ~0.978 | ~0.396 | ~0.627 | Conservative HA-LW classification |
| Youden index | 0.133 | 0.810 | 0.534 | 0.364 | 0.753 | ~0.750 | ~0.491 | ~0.751 | Higher recall but lower precision |
